# Diagnostic analysis of lupus anticoagulant using clot waveform analysis in activated partial thromboplastin time prolonged cases: A retrospective analysis

**DOI:** 10.1002/hsr2.258

**Published:** 2021-03-12

**Authors:** Kazunori Kanouchi, Hiroto Narimatsu, Toru Shirata, Keita Morikane

**Affiliations:** ^1^ Division of Clinical Laboratory Yamagata University Hospital Japan; ^2^ Cancer Prevention and Control Division Kanagawa Cancer Center Research Institute Yokohama Japan; ^3^ Graduate School of Health Innovation Kanagawa University of Human Services Kawasaki Japan; ^4^ Department of Genetic Medicine Kanagawa Cancer Center Yokohama Japan

**Keywords:** activated partial thromboplastin time, antiphospholipid antibody syndrome, clot waveform analysis, lupus anticoagulant

## Abstract

**Background and Aims:**

Hemophilia was diagnosed in precedence research of clot waveform analysis (CWA) using the activated partial thromboplastin time (APTT). In patients with antiphospholipid syndrome (APS), lupus anticoagulant (LA) causes an increase in APTT, suggesting that the waveform would probably be distorted. Therefore, we evaluated using clinical samples. CWA may be useful low cost for clinical detection of LA. We assessed the clinical value of CWA for detection of LA and coagulation using clinical blood samples collected from patients with a prolonged APTT.

**Methods:**

We used patient samples inspected between April 2011 and March 2013 in Yamagata University Hospital. CWA was performed using the ACL TOP coagulation analyzer, and the associated software program was used to calculate APTT clotting endpoints. An atypical peak was defined as a derivative plot that did not conform to the normal S‐shaped clot reaction curve.

**Results:**

In total, 162 patients, including 66 men and 96 women, with an average age of 46 years (range: 24‐89 years) were included. We also collected control samples from unmatched healthy donors. All 162 patients were divided according to medication history or condition into the following five groups: heparin (n = 20), warfarin (n = 23), hepatic dysfunction (n = 13), normal (n = 20), and LA‐positive antiphospholipid syndrome (APS; n = 86). Twenty healthy individuals were included as controls.

Eighty patients had an atypical peak. Among all, 78 patients (90.6%) were LA‐positive, and 2 patients (2.5%) were treated with warfarin. The remaining two patients had prothrombin time international normalized ratios (PT‐INR) >4.0 while taking warfarin. Those who were APS LA positive with thrombosis and without thrombosis had split the reaction of clotting time, deceleration/acceleration time (D/A) ratio of 2.36 (1.99,3.24) vs 2.34 (2.04,2.86), respectively.

**Conclusion:**

The significant atypical peak and D/A ratio extension may be explained by the clotting waveforms observed specifically in patients with LA‐positive APS.

## 1 INTRODUCTION

Antiphospholipid syndrome (APS) is a systemic autoimmune disease characterized by the presence of antiphospholipid antibodies and thrombophilia, and it is associated with morbidity during pregnancy.^1^ A definitive diagnosis of APS is made depending on both clinical and laboratory findings. Clinical findings include thrombosis or pregnancy‐related complications such as spontaneous abortion, fetal death, or eclampsia. Laboratory findings include the detection of antiphospholipid antibodies, lupus anticoagulant (LA), anticardiolipin IgG or IgM antibodies, or anti‐β2‐glycoprotein I antibodies.^2^

LA is measured using the dilute Russell's viper venom time (dRVVT) test or the STACLOT LA assay, which is based on the activated partial thromboplastin time (APTT). Both tests require expensive equipment and reagents. In contrast, a less expensive APTT method is used commonly at many hospitals.^2^, ^3^

Recently, the development of a fully automated coagulation‐measuring device, the ACL TOP (Instrumentation Laboratory: IL, Lexington, KY, USA) and the associated software program enabled clinicians to conduct a waveform analysis of patients with coagulation disorders. Waveform analysis is generally low cost, and a lot of information can be obtained by from APTT.

In this analysis, the optical solidification reaction waveform curve (ie, clotting curve) can be visualized using absorbance measurements.^4^, ^5^, ^6^, ^7^, ^8^, ^9^ Complex waveform analyses, which can be performed using ACL TOP software, have been used to evaluate the prognoses of patients with disseminated intravascular coagulation (DIC) and hemophilia.^10^, ^11^, ^12^, ^13^, ^14^, ^15^, ^16^, ^17^

In patients with APS, LA induces abnormal extension of the APTT, which suggests that the waveform would probably be distorted. Therefore, clot waveform analysis (CWA) may be a useful low‐cost alternative for the clinical detection of LA. In this study, we assessed the clinical value of the CWA for detecting LA in blood samples from patients with various blood coagulation disorders and those treated with warfarin and heparin at a tertiary care university hospital in Japan.

## 2 MATERIALS AND METHODS

### 2.1 Samples

Yamagata University Hospital is a tertiary acute care hospital with departments that provide specific medical care to patients with autoimmune diseases such as systemic lupus erythematosus and APS, hematologic diseases such as coagulation disorders, and obstetric and gynecological diseases.

This was a retrospective study undertaken between April 2011 and March 2013. After patient samples were collected, we prospectively collected unmatched health donor samples. Informed consent was obtained from each of the healthy donors. A total of 142 blood samples were obtained from 142 patients who underwent prothrombin time (PT) and APTT assessments. Blood samples were collected from 20 healthy staff members with a normal PT and APTT and no history of medication use (8 men and 12 women with an average age of 45 years and age range between 24 and 62 years) who were unmatched during the study period. The study protocol and procedures used for handling samples were in accordance with the procedures approved by the Institutional Review Board of Yamagata University Faculty of Medicine (approval nos. H19‐141 and H27‐299). The researchers adhered and agreed closely to all approved protocols. Informed consent was obtained from each of the participants. According to our preliminary study with a small sample size, the expected specificity of the CWA was 80% in patients of LA‐positive samples. Assuming that the range of a 90% confidence interval (CI) was 10%, 132 patients' samples were adequate to achieve a power of 80%, with an alpha error of 0.05. According to the distribution of the cases from the Yamagata University Hospital, 3 years of collection were required.

### 2.2 Methods of measurement

One hundred forty‐eight patients whose blood samples indicated prolonged PT and APTT were divided into the following six groups. The first group comprised 86 LA‐positive patients, we numbered the patients with APS consecutively, and two samples were collected per person, those patients whose samples were included had a clinically confirmed diagnosis by the doctors.”

The second group comprised 20 patients, who received unfractionated porcine heparin therapy (1000‐2000 units/d) and whose APTT values were within our therapeutic range (47‐77 seconds) (Table 1). These patients had been admitted to the cardiac, renal, and vascular wards and were receiving treatment for conditions such as acute coronary syndrome, myocardial infarction, and deep vein thrombosis/pulmonary embolism (DVT/PE). The third group comprised 23 patients who had DVT/PE and cerebrovascular diseases who were treated with warfarin (10‐20 mg).^18^ The fourth group included 13 patients who had liver disease and met the diagnostic criteria of impaired liver function. The patients were diagnosed according to the most recent guidelines established by the American Association for the Study of Liver Diseases (AASLD).^19^

**TABLE 1:** Results of LA tests of LA‐positive and LA‐negative patients included in this study

Patients	Number	PT‐INR^a^	APTT(S)^a^	Screen ratio^a^	DRVVT ratio^a^	STACLOT®(S)^a^
Normal	20	1.02 ± 0.04	32.5 ± 3.5	1.02 ± 0.3	1.05 ± 0.1	0.5 ± 2.5
Warfarin	23	3.05 ± 0.63	34.0 ± 3.7	1.08 ± 0.5	1.09 ± 0.1	2.1 ± 1.9
Liver disease	13	2.89 ± 0.53	39.0 ± 6.7	1.03 ± 0.7	1.02 ± 1.1	3.1 ± 2.3
Heparin	20	1.91 ± 0.02	62.1 ± 15.3	1.09 ± 0.5	1.02 ± 0.2	3.6 ± 2.2
APS LA(+) of thrombosis(+)	33	2.84 ± 0.38	58.2 ± 12.4	1.18 ± 0.2	1.58 ± 1.2	19.8 ± 10.4
APS LA(+) of Thrombosis(−)	53	1.09 ± 0.04	60.1 ± 23.1	1.17 ± 0.1	1.75 ± 0.8	18.3 ± 9.3
Reference range		0.9‐1.1	28.0‐36.0	>1.15	> 1.3	> 8.0

Abbreviations: APS, antiphospholipid antibody syndrome; APTT, activated partial thromboplastin time; D/A, deceleration time/acceleration time ratio; DRVVT, dilute Russell viper venom time; heparin, the patient in a 5000 units of heparin(s) medication treatment; LA, lupus anticoagulant; normal, normal patients–plasma; PT‐INR, prothrombin time‐international normalized ratio; reference range, manufacturer's cut off value; screen ratio, DRVVT screen (patient plasma/pooled normal plasma) $\text{DRVVT ratio}=\frac{\left\{\left(\frac{\text{patient plasma}}{\text{pooled normal plasma}}\right)\;\text{screen ratio}\right\}}{\left\{\left(\frac{\text{patient plasma}}{\text{pooled normal plasma}}\right)\;\text{confirm ratio}\right\}};$
STACLOT®, LA measurement kit marketed by Roche; warfarin, the oral anticoagulant therapy by warfarin (INR:1.5‐3.2).

^a^ Mean ± 2 SD.

From all patients, a 1.8‐mL blood sample was collected into a tube containing 0.109 mol/L trisodium citrate (Venoject II, Terumo Company, Tokyo, Japan). Blood samples were centrifuged at 2000 *g* and approximately 23°C for 15 minutes. Plasma was separated from each sample and transferred to a fresh collection tube, followed by centrifugation at 2800 *g* and 23°C for 15 minutes. This double‐centrifugation process renders the plasma platelet‐poor (final platelet count <1 × 10^9^/L), an essential characteristic for LA detection. All samples were freshly obtained on the day of assessment. The samples were assessed for PT and APTT as described previously,^20^ using the ACL TOP 750 automated coagulation device [Instrumentation Laboratory (IL), Lexington, KY, USA]. APTT‐SP (IL, Tokyo, Japan) was used for APTT testing, while recombinant tissue thromboplastin (RecombiPlasTin 2G, ISI 0.99; IL) was used to assess PT and calculate the international normalized ratio. The dRVVT was performed using LA Screen (Life Therapeutics, Sydney, NSW, Australia). LA was also detected using the hexagonal phospholipid neutralization test according to the APTT method and using the STACLOT LA (Diagnostica Stago, Asnieres‐Sur‐Seine, France) measuring kit. STACLOT LA was also analyzed using STart 4 (Young Instruments, Stago, France) automated coagulation analyzer according to the manufacturer's instructions. We performed measurements according to LA guidelines^2^, ^3^ on evaluation of the effectiveness of the CWA.

### 2.3 Clot waveform analysis (CWA)

We conducted the CWA using the APTT method, as described by Solano et al.^9^ Briefly, 0.1 mL of surface‐active agents and 0.1 mL of calcium chloride were added to 0.1 mL of double‐centrifuged plasma. The time required for sample solidification due to fibrin formation indicates the completion of coagulation. We used the initial change in light permeability resulting from the conversion of fibrinogen to fibrin paste to determine the start of coagulation and constructed the coagulation waveform from this point. These curves were a graphical representation of the optical data recorded during clotting tests such as APTT. While the mathematical algorithms used to generate the clotting endpoint vary with different optical endpoint analyzers, all clot reaction curves contain the same elements—a plot of absorbance or light transmission or light scatter units vs acquisition time to produce a curve.^9^

**FIGURE 1:**
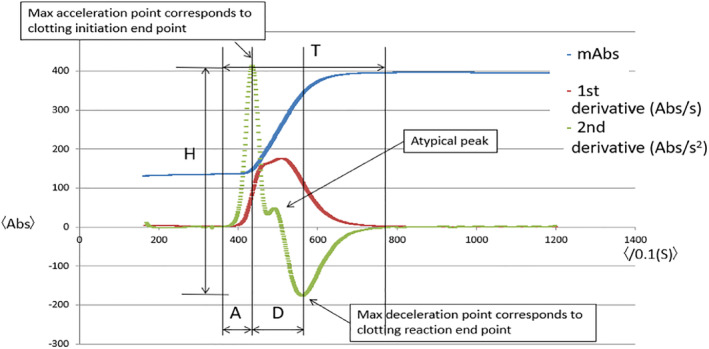
A clot waveform analysis. An example plot of the activated partial thromboplastin time (APTT) in normal plasma. The typical peak clot reaction curves are illustrated

The software program on the ACL TOP analyzer allows display of the clot reaction curves (mAbs) and the associated first and second derivative curves in (Figure 1). These derivative plots are automatically calculated using the ACL TOP software program from absorbance data and reflect the velocity (1st derivative [Abs/s]) and acceleration and deceleration (2nd derivative [Abs/s^2^]) at various points throughout the clotting reaction, which is depicted as the clot reaction curve, as indicated by the blue, red, and green lines, respectively, in the example (Figure 1). The inflection point of the 2nd derivative point (peak acceleration point) defines the clotting endpoint (Figure 1). These curves represent the primary mathematical algorithm used by the ACL TOP software to calculate the APTT clotting endpoints date.^9^ The ACL TOP 750 utilizes a light scatter principle, and the ACL TOP 750 software split the reaction time into two phases. This was the primary mathematical algorithm used by the ACL TOP software to calculate APTT clotting endpoints. Using the acceleration curve, we then split the reaction time into two phases, acceleration time (A) and deceleration time (D), which were reported in seconds. We defined A as the time from the reaction initiation to the peak of acceleration, and D as the time from the peak of acceleration to the lowest absorbance. We defined height (H) as the difference between the absorbances of the maximum peak of acceleration and the lowest value. We also defined the total reaction time (T) as the time from the beginning to the completion of the reaction (Figure 1). Solano et al characterized the atypical derivative plot pattern as a distinctly biphasic plot associated with a double peak on the second derivative plot. The second derivative plots associated with this shoulder‐like peak are termed the atypical peak.^9^ An example of this pattern is illustrated in (Figure 1). We used EZR software on R commander (version 2.13.0) to conduct the statistical analysis.^21^ We used the Kruskal‐Wallis method to assess the significance of the presence of atypical peaks with respect to the median (interquartile range) of A, D, D/A, H, and T.

## 3 RESULTS

The first group comprised 86 LA‐positive patients, including 24 men and 62 women, with an average age of 43 years (range: 24‐73 years), who met the diagnostic criteria of international consensus statements regarding updates of the classification criteria for definite antiphospholipid syndrome (APS).^1^ The 86 APS‐positive patients included 33 patients with a history of thrombosis and 53 patients without anticoagulation or a history of thrombosis anticoagulation. All patients had both prolonged PT and APTT. A history of thrombosis was present in 33 patients, and 33 patients received anticoagulant therapy. The second group comprised 20 patients, including 14 men and 6 women with an average age of 56 years (range: 34‐78 years). The third group comprised 23 patients, including 12 women and 11 men with an average age of 65 years (range: 24‐89 years) (Table 1). The fourth group included 13 patients, including 8 women and 5 men with an average age of 65 years (range: 31‐80 years) (Table 1).

Table 2 presents the mean A, D, D/A, H, and T values and the presence of atypical peak according to the groups of patients stratified by participants grouped by condition (normal, warfarin, hepatic dysfunction, heparin, and APS LA‐positive). An atypical peak was observed in 80 of all 168 (47.6%) cases and in 78 of 86 (90.7%) APS LA‐positive cases. Among non‐LA individuals, atypical peak was observed in 2 of 82 cases (2.3%), and both patients had a PT‐INR >4.0 and were taking warfarin. A comparative analysis of the measured parameters across all groups indicated that the extension of the deceleration time was most significant in the LA group (*P* = 0.026).

**TABLE 2:** LA test results for LA‐positive and LA‐negative patients used in this study and CWA results

Patients	Number	Atypical peak	Acceleration time^a^	H^a^	Deceleration time^a^	T^a^	D/A ratio^a^
Normal	20	0	48.1 (42.1,54.9)	1046.7 (732.51311.5)	96.7 (80.4107.3)	357.5 (315.2399.5)	1.98 (1.86,2.11)
Warfarin	23	2	58.6 (33.1,87.1)	713.2 (563.2972.3)*	102.2 (67.6163.2)	523.7 (434.8724.5)*	1.58 (2.21)
Hepatic dysfunction	13	0	52.1 (33.1,75.5)	686.2 (521.3856.2)*	89.6 (73.2132.5)	475.8 (393.2575.9)	1.81 (1.59,2.02)
Heparin	20	0	70.1 (52.6,89.1)*	834.5 (602.31163.8)	121.2 (92.4143.2)	464.5 (375.9547.2)	1.61 (1.43,1.96)
APS LA(+) of thrombosis(+)	33	29	92.1 (60.2141.1)*	473.3 (358.5622.5)*	177.4 (87.4277.4)*	714.5 (623.1837.2)*	2.36 (1.99,3.24)*
APS LA(+) of thrombosis(−)	53	49	104.1 (58.1165.1)*	418.1 (355.7788.4)*	253.1 (146.3253.2)*	697.3 (291.0,1021.2)*	2.34 (2.04,2.86)*
Reference range			>54.1	>1189.5	>102.5	>397.0	>2.10

Abbreviations: APS, antiphospholipid antibody syndrome; APTT, activated partial thromboplastin time; D/A, deceleration time/acceleration time ratio; DRVVT, dilute Russell viper venom time; heparin, the patient in a 5000 units of heparin(s) medication treatment; LA, lupus anticoagulant; LA judgment, The presence of LA was confirmed by a positive result in STACLOT LA or the dilute Russell viper venom time (DRVVT) which is considered as the gold standard; normal, normal patients –plasma; reference range; 95% of a healthy person's section width; manufacture's cut off value; STACLOT®, LA measurement kit marketed by Roche; warfarin, the oral anticoagulant therapy by warfarin (INR:1.5‐3.2).

^a^ Variables are presented as median (IQR).

* *P* < .05 compared with normal.

## 4 DISCUSSION

We demonstrated the presence of an atypical peak, an extended CWA waveform, and significantly extended D and T values in APS LA‐positive patients and those on warfarin relative to the control group (Table 2).

It indicated that the extended deceleration time in a clotting waveform might be specific to LA‐positive APS and was caused by the onset of the atypical peak. For future studies, these two findings may be valuable for differentiating LA‐positive APS from other coagulation abnormalities for which the basic mechanism underlying the onset of atypical peaks remains unclear. Further research with a larger study population is warranted.

Our findings suggest a potential hypothesis regarding the mechanism of onset of the atypical peak, namely, that the atypical peak may be induced by blocking the conversion of fibrinogen to fibrin during the coagulation process. Evidence supporting this hypothesis includes the high PT‐INR (>4.0) observed in the two patients with an atypical peak who were treated with warfarin, which indicated low levels of prothrombin. Prothrombin is the precursor of an enzyme converting fibrinogen to fibrin, and it may also form a complex with LA and a major phospholipid binding protein. In this study, limited plasma volumes precluded the measurement of prothrombin. In future research, both prothrombin and fibrin levels should be measured and investigated to determine whether either variable is associated with the atypical peak. Reportedly, anti‐PS/prothrombin IgG antibodies (aPS/PTs) were frequently detected in patients with APS. In these patients, prothrombin activity was decreased by phospholipid‐binding protein to prothrombin. In this study, the number of patients with a high value for PT‐INR and prothrombin activity may have decreased, and the unusual waveform of APTT was possibly due to the low level of prothrombin activity.^22^, ^23^ The analysis of various mixtures of prothrombin‐deficient and normal plasma in different ratios would enable an examination of changes in atypical peak. An experiment that artificially reproduces the atypical peak of CWA could further confirm the diagnostic value of CWA for LA‐positive APS. In this study, we surmised that CWA based on a solidification waveform determined using the APTT method could provide useful clinical information for patients with other diseases in which LA could be distinguished by an extension of the APTT.^9^, ^24^ However, the mechanism by which LA causes an extended deceleration of the clotting waveform requires further study. However, 29 of 33 patients received anticoagulant therapy, and the anticoagulant therapy may have affected the results. We did not measure the C‐reactive protein and cholesterol levels, as these were not known to influence abnormal clot waveforms.

In conclusion, our data suggest that the CWA observed conventional tests for LA. As shown in Table 2, however, the atypical peak appeared highly LA‐positive, and the CWA of data obtained in a previous study was different from that in the present study.^9^ Although a previous study also involved CWA using an ACL TOP analyzer, the authors reported that the atypical peak was associated not only with LA but also with FVIII and FIX deficiency.^11^ Moreover, the variation in the appearance and features of atypical peaks varied according to APTT reagents and the presence of LA, FVIII deficiency, or FIX deficiency, when measured using the analyzer. Finally, previous findings regarding the atypical peak are limited to CWA using ACL TOP analyzers, as in the present study. Further studies should explore whether an atypical peak also appears when using other analyzers.

## CONFLICT OF INTEREST

The authors declare no potential conflicts of interest associated with this manuscript.

## AUTHOR CONTRIBUTIONS

Conceptualization and design: Kazunori Kanouchi and Hiroto Narimatsu

Provision of study materials or patients and Methodology: Kazunori Kanouchi, Hiroto Narimatsu, Tooru Shirata, and Keita Morikane

Collection and assembly of data curation and Investigation: Kazunori Kanouchi and Hiroto Narimatsu

Formal data analysis and interpretation: Kazunori Kanouchi and Hiroto Narimatsu

Project Administration and Supervision: Kazunori Kanouchi and Hiroto Narimatsu

Manuscript writing original draft preparation: Kazunori Kanouchi, Hiroto Narimatsu

Final approval of the manuscript and Validation: Kazunori Kanouchi, Hiroto Narimatsu, Tohru Shirata, and Keita Morikane

Writing review and editing: Kazunori Kanouchi

Corresponding author and manuscript guarantor: Hiroto Narimatsu

Funding Acquisition: None

 All authors have read and approved the final version of the manuscript. Hiroto Nariamtsu had full access to all of the data in this study and takes complete responsibility for the integrity of the data and the accuracy of the data analysis.

 All authors have read and approved the final version of the manuscript. Corresponding author had full access to all of the data in this study and takes complete responsibility for the integrity of the data and the accuracy of the data analysis.

## TRANSPARENCY DECLARATION

We affirms that this manuscript is an honest, accurate, and transparent account of the study being reported; that no important aspects of the study have been omitted; and that any discrepancies from the study as planned (and, if relevant, registered) have been explained.

## Data Availability

The data that support the findings of this study are available on request from the corresponding author. The data are not publicly available due to privacy or ethical restrictions.
